# Barriers to adopting therapeutic virtual reality: the perspective of clinical psychologists and psychotherapists

**DOI:** 10.3389/fpsyt.2025.1549090

**Published:** 2025-03-18

**Authors:** Anna Felnhofer, Franziska Pfannerstill, Lisa Gänsler, Oswald D. Kothgassner, Elke Humer, Johanna Büttner, Thomas Probst

**Affiliations:** ^1^ Department of Pediatrics and Adolescent Medicine, Division of Pediatric Pulmonology, Allergology and Endocrinology, Comprehensive Center for Pediatrics, Medical University of Vienna, Vienna, Austria; ^2^ Division of Psychotherapy, Department of Psychology, Paris Lodron University Salzburg, Salzburg, Austria; ^3^ Faculty of Psychology, University of Vienna, Vienna, Austria; ^4^ Department of Child and Adolescent Psychiatry, Comprehensive Center for Pediatrics, Medical University of Vienna, Vienna, Austria; ^5^ Faculty for Psychotherapy Science, Sigmund Freud University Vienna, Vienna, Austria; ^6^ Department for Psychosomatic Medicine and Psychotherapy, University for Continuing Education Krems, Krems, Austria

**Keywords:** clinical psychology, psychotherapy, implementation, barriers, virtual reality

## Abstract

**Background and objective:**

Despite evidence supporting the effectiveness of Virtual Reality (VR) for mental disorders, VR adoption in therapy remains low. As VR-technology continues to advance, it is crucial to examine individual and contextual barriers preventing implementation of therapeutic VR.

**Methods:**

An online survey with closed and open-ended questions regarding knowledge of VR, VR-usage and barriers to VR adoption was conducted among clinical psychologists and psychotherapists in Austria (M_age_=51.71 years, 76% women).

**Results:**

Of 694 participants, only 10 reported using therapeutic VR. Chi-square tests revealed significant differences regarding interest in therapeutic VR based on prior experience, employment status, professional training, and therapeutic cluster. Besides a small age effect, no effects of gender or professional experience were found. Participants interested in VR (interest group, IG) frequently cited barriers and other reasons (see thematic analysis) for not having used VR yet. Those not interested in VR (no interest group, NIG) indicated a lack of relevance, no perceived advantage, or disinterest as reasons for not using VR. Thematic analysis identified four themes shared by both IG and NIG, each encompassing group-specific sub-themes: *professional barriers* (lack of knowledge, training, time, personal reasons), *financial barriers* (costs, cost-benefit-ratio), *therapeutic barriers* (clinical applicability, concerns about “real” therapeutic relationship), and *technological barriers* (immature technology, cybersickness, no equipment).

**Conclusions:**

Significant barriers to the adoption of therapeutic VR among clinical psychologists and psychotherapists are gaps in knowledge and training, financial constraints, and lack of motivation, all of which highlight the need for training and financial support to enhance VR implementation.

## Introduction

Over the past decades, immersive Virtual Reality (VR) has emerged as a promising tool for treating a variety of mental disorders (1). Given the inconsistent use of the term “VR” in literature, it is essential to specify that, in the current paper, “VR” refers to fully immersive 3D systems. This involves a computer-simulated, interactive, three-dimensional environment experienced through a Head-Mounted Display (HMD), which provides a high-fidelity multisensory experience, a 360°field of view, and movement tracking creating a sense of immersion for users (2). In this context, immersiveness refers to the characteristics of the VR technology, ranging on a continuum from low (e.g., 2D screens) to high (e.g., 360°HMDs) immersion (3). A key feature of immersive VR, which likely contributes to its therapeutic effectiveness (4), is its ability to elicit a sense of presence – i.e., the impression of being in the virtual environment (5) – allowing for realistic, life-like responses (6). As VR offers a safe, controlled, customizable, and potentially scalable environment that approximates the sensory richness and contextual detail of real-world experiences (2, 7), it constitutes a promising tool for broad therapeutic applications.

Traditionally, VR has most commonly been used in the form of VR exposure therapy (VRET) for anxiety disorders. Numerous high-quality randomized controlled trials have demonstrated its efficacy, with effect sizes and attrition rates comparable to those of *in-vivo* exposure (8–11). As a result, VRET has been integrated into clinical treatment guidelines, particularly for specific phobias (12). Beyond anxiety disorders, VR has also shown strong evidence for posttraumatic stress disorder (PTSD, 1, 13) and, with less robust support, for conditions such as substance use disorders (14), eating disorders (15), and schizophrenia spectrum disorders (16, 17). Additionally, VR shows promise for augmenting adjuvant treatments such as biofeedback (18, 19) and cognitive training in dementia (20). Although most research has focused on adults, early studies on children and adolescents – particularly in the treatment of autism (21) and anxiety (22) – have yielded similarly promising results. VRET may be considered a valuable addition to therapists’ and clinical psychologists’ therapeutic tools. While it demonstrates effectiveness comparable to traditional *in-vivo* exposure therapy (8–11), it offers key advantages, including greater cost-effectiveness, increased accessibility, and enhanced standardization and scalability (23, 24). Furthermore, *in-vivo* exposure is not always feasible – such as with personal traumatic stimuli (25) or hard-to-access stimuli like thunderstorms, flying, animals, or crowds (26). As such, VRET provides an alternative that was previously impractical, particularly in clinical settings (27). Moreover, two studies found that 76%-89% of participants preferred VRET over traditional *in-vivo* exposure therapy (28, 29).

In addition to these advantages, the release of commercial HMDs like the Oculus Rift in 2016 ushered in a new generation of accessible, affordable, and high-quality VR hardware, enhancing its availability to mental health professionals (26). However, despite this increased accessibility and the growing body of supporting evidence (1), data suggests that VR remains largely confined to research and specialized clinical settings, with limited adoption in mainstream treatment settings (26, 27, 30, 31). This slow integration mirrors the broader trend in evidence-based treatments for mental disorders, which typically take more than a decade to be fully implemented in routine practice (32, 33). To accelerate the implementation of evidence-based VR therapy, there is a need to identify and address both individual and contextual barriers that impede the widespread adoption of VR in everyday mental health care.

To date, few studies have explored the adoption of new-generation HMDs. Those that do suggest that past concerns, which primarily centered around hardware issues (e.g., bulkiness of equipment and low graphical quality), may now have shifted towards a more complex interplay of individual and contextual factors (26, 30, 34). Among individual barriers, negative attitudes toward VR, limited knowledge of VR, and concerns about its safety and efficacy are commonly reported; contextual barriers, in turn, include logistical challenges, resource constraints, and technological limitations (26, 30, 34). However, much of the existing research is limited by small sample sizes (30, 34), a narrow focus on specific groups – such as cognitive-behavioral therapists (26) – or specific applications like rehabilitation (35), thereby restricting the generalizability of findings to other therapeutic approaches, patient populations, and clinical settings.

Despite VR’s growing accessibility and proven efficacy, its adoption in mental health care remains limited due to individual and contextual barriers. Existing research, often constrained by small samples or narrow focus, provides little insight into the broader adoption of VR across diverse clinical settings and therapeutic backgrounds. Therefore, this study aims to assess the rate of VR adoption among a large sample of practicing Austrian clinical psychologists and psychotherapists from various therapeutic orientations and employment conditions. In Austria, both psychotherapists and clinical psychologist are qualified to diagnose and treat patients with mental disorders using non-medical interventions. Clinical psychologists are required to complete a master’s degree in psychology, followed by specialized clinical psychologist training. Psychotherapists, on the contrary, must be trained in one of the 23 psychotherapy methods accredited in Austria, and entering psychotherapy training is possible for various professional qualifications. In addition to investigating VR adoption, this study aimed to explore differences between those interested in using VR and those who are not. By identifying individual and contextual barriers to VR adoption, this study has the goal of informing strategies to promote VR adoption and facilitate the sustainable transfer of evidence-based VR practices into clinical practice.

## Materials and methods

The reporting of this study followed the Strengthening the Reporting of Observational Studies in Epidemiology (STROBE, 36) guidelines for quantitative analyses, and – where applicable – the Consolidated criteria for reporting qualitative research (COREQ, 37) for qualitative data.

### Participants and procedure

For the study, eligible participants were all licensed Austrian psychotherapists and clinical psychologists registered in the list of psychotherapists and/or in the list of clinical psychologists of the Austrian Federal Ministry of Social Affairs, Health, Care and Consumer Protection. This study utilized a cross-sectional online survey implemented in LimeSurvey (38), which was opened between May 29, 2024, and June 22, 2024. The survey took about 30 minutes for participants and consisted of 170 items on various topics, but only sociodemographic and professional characteristics and VR-related questions were analyzed for this study. Some items could be skipped if they were not relevant or applicable to individuals. The survey was distributed to psychotherapists and clinical psychologist via a link sent by e-mail. Participants were contacted if a valid e-mail address was recorded in the official professional list of psychotherapists or clinical psychologists (20,830 accessible participants) with the support of psychotherapy institutions cooperating with the universities of the authors. At the beginning of the survey, participants had to read the study information and their active consent to this information and to the data protection declaration was obtained by pressing/clicking the field “I agree” (electronic informed consent). No incentives were provided, and participation was voluntary. The study was reviewed and approved by the ethics committee of the Paris Lodron University of Salzburg (protocol number GZ 20/2024). A total of 694 Clinical Psychologists and Psychotherapists aged 27–83 years (M_age_=51.71 ± 11.11 years, 75.8% women, 0.9% diverse, 23.3% men) completed the survey (completion rate of 53.3%). Participation was voluntary and no incentives were offered.

### Measures

#### Social and professional demographics

Participants were asked about their gender, age, and details regarding their therapeutic or clinical practice, including their employment status (employed, self-employed, or both), the age groups of the patients they treat, and their professional training (whether they are trained as a psychotherapist, clinical psychologist, or both). Additionally, they were asked to provide the year of their registration in the official registry of psychotherapists and/or clinical psychologists, as well as their psychotherapeutic method [23 psychotherapeutic methods are accredited in Austria which can be grouped into four clusters (psychodynamic, humanistic, systemic, and behavioral; 39)].

#### VR-usage, interest, and opinion

For VR-related items used in this study, see **Supplementary Material A**. All participants were asked whether they used VR in treatments (“Do you use virtual reality (VR) in your treatments (via VR glasses, e.g. Meta Quest)?”). Apart from mentioning VR-glasses, “VR” was not further specified. Depending on the answer to this question, different sub-surveys followed. Participants who responded with yes were asked about the age group of their VR-patients and what disorder(s) they used VR for. Also, they were asked the following questions (including free-text response options): (1) “In your opinion, what are the advantages of using VR? – for you?/for the patients?”, (2) “What problems does it cause? – for you?/for the patients?”, (3) “What would you wish for the implementation of VR in treatment?”.

Participants who had not used VR in treatment yet were asked, whether they have tried VR and whether they would like to use it in treatment (see **Supplementary Material A**). If they were interested, they could select several answers in a multiple choice question (e.g., “I am not sure for which patients VR is beneficial”), and/or provide free-text responses to the following items: (1) “I have reservations about VR”, (2) “There are barriers that make it impossible for me to use it”, and (3) “Other reasons”. If participants were not interested in using VR, they again selected among multiple-choice responses (e.g., “Do not see any benefit for me/for my patients”), and/or answered the same free text-items as those who were interested in using VR.

### Data analyses

#### Quantitative

Analyses were conducted using IBM SPSS 29 (40). Descriptive statistics were applied for the group of mental health professionals who already use VR in treatments as this group includes only ten participants. Chi-square tests were used to examine differences in sociodemographic and professional characteristics between mental health professionals who do not use VR but are either interested (interest group, IG) or not interested (no interest group, NIG) in using VR for treatment. Additionally, a t-test for independent samples was used to assess the age difference between these two groups. Multiple-choice questions about reasons for not using VR in treatments were analyzed using descriptive statists for both IG and NIG.

#### Qualitative

Inductive content analysis (41, 42) was applied without a pre-existing framework or coding scheme to both groups: those stating to use VR in treatment and those not using it. For participants already working with VR, we focused on the following free-text responses in our thematic analysis: (1) “In your opinion, what are the advantages of using VR? – for you?/for the patients?”, (2) “What problems does it cause? – for you?/for the patients?”, (3) “What would you wish for the implementation of VR in treatment?”.

For participants not using VR, we focused on the question “Would you like to use VR for your treatments?” and on the two resulting groups, answering “no” (NIG) or “yes” (IG). Our thematic analysis was then conducted separately for these two groups, analyzing free-text responses to the questions “Why haven’t you been able to use VR yet?” (IG) and “What are the reasons for not wanting to use VR?” (NIG), across the three free-text items: “I have reservations about VR so far”, “There are barriers that make it impossible for me to use it” and “Other reasons”.

The first step of the thematic analysis involved an in-depth familiarization with the data. To ensure the reliability of the qualitative coding process, two independent coders (LG and AF) analyzed a subset of responses (10%) to establish a coding framework. These initial codes were generated to capture key topics, which were subsequently reviewed, compared between the two coders, and grouped into broader themes and sub-themes. Discrepancies were discussed and resolved through consensus in an iterative process, incorporating regular meetings and ongoing revisions to minimize interpretation bias. After achieving satisfactory agreement (i.e., Cohen’s Kappa with values above 0.70), the remaining data were coded by the two coders, with periodic checks to maintain consistency. If one free-text answer contained several different topics, the answer was duplicated and allocated to each relevant theme.

## Results

### Characteristics of VR users

Of 694 participants, only 1.4% of surveyed participants (n=10, M_age_=46.30 ± 8.97 years, range: 31–57 years) reported using VR in their practice (see **Table 1**), while 20.5% reported an interest to use it and 79.5% were not interested in using it. Half of VR users were psychotherapists, and half stated to be self-employed. Most (70%) used VR primarily with adults, though one therapist used it with children. The primary conditions treated with VR were anxiety disorders (80%), with 40% using VR for depression and PTSD. Considering the small sample size, thematic analyses were limited. VR users reported several advantages of using therapeutic VR, with overlapping answers regarding advantages for oneself and for patients, including expanded therapeutic options, such as improved access to therapy, tailored environments, flexible exposure settings, and immediate stimulus generation. VR was seen as secure, motivating, offering novel, engaging experiences. However, problems were also noted, again with overlapping themes for oneself and patients, such as high technical demands, additional preparation time, underdeveloped technology, image glitches or device failures, lack of provider support, cybersickness, and the risk of patients avoiding exposure (i.e., by closing their eyes) without the therapist’s awareness. Regarding wishes for therapeutic VR implementation, VR users expressed a need for more research and development, user-friendly programs approved by health insurance, and better-quality software.

**Table 1:** Characteristics of VR-users (n=10) among Austrian clinical psychologists and psychotherapists.

Age, M (SD)	46.30 (8.970)
Gender, n (%)	
Women	5 (50)
Men	4 (40)
Diverse	1 (10)
Employment Type, n (%)	
Self-employed	5 (50)
Employed	5 (50)
Both	0 (0)
Training, n (%)	
Clinical Psychology	5 (50)
Psychotherapy	5 (50)
Cluster: behavioral	2 (20)
Cluster: systemic	1 (10)
Cluster: humanistic	2 (20)
Cluster: psychoanalytical-psychodynamic	0 (0)
VR use with…, n (%)	
Adults only	7 (70)
Children only	1 (10)
Both	2 (20)
VR use for… (multiple answers), n (%)	
Anxiety disorders	8 (80)
Depression	4 (40)
PTSD	4 (40)
Relaxation	2 (20)
Dementia	1 (10)
Addiction	1 (10)
Somatic disorders (unspecified)	1 (10)
Social skills training	1 (10)

PTSD, posttraumatic stress disorder.

### Group differences between non-users (interest group IG vs. no interest group NIG)

Chi-square tests revealed significant differences between IG (n=140, 20.5%) and NIG (n=544, 79.5%) in variables such as prior experience with VR usage, employment status, professional training, and therapeutic cluster, but no significant differences were found for gender and years of professional experience (see **Table 2**). Those who had experienced VR themselves before, and professionals who are either employed or hold both self-employment and employment roles were more likely to express interest than mental health professionals who had not yet experienced VR or are solely self-employed. Clinical psychologists and professionals with both clinical psychology as well as psychotherapy training indicated more interest in VR usage for treatment than psychotherapists. A strong relationship emerged across theoretical orientations within psychotherapists, with behavioral therapists showing the highest interest compared to therapists from the psychoanalytical-psychodynamic, humanistic or systemic cluster.

**Table 2:** Crosstabulation of the group differences between those interested in using VR and those who are not interested.

	Interest in VR adoption		Test statistics		*p*
	IG (n=140)	NIG (n=544)			
**Experience with VR, n (%)**			χ² = 11.81	Φ = 0.13	< 0.001
Yes	76 (54.3)	208 (38.2)			
No	64 (45.7)	336 (61.8)			
**Employment type, n (%)**			χ² = 7.80	Φ = 0.11	0.020
Self-employed	85 (60.7)	396 (72.8)			
Employed	10 (7.1)	28 (5.1)			
Both	45 (32.1)	120 (22.1)			
**Training, n (%)**			χ² = 12.94	Φ = 0.14	0.002
Psychotherapy	66 (47.1)	347 (63.8)			
Clinical Psychology	43 (30.7)	117 (21.5)			
Both	31 (22.1)	80 (14.7)			
**Therapeutic Cluster (n = 515), n (%)**			χ² = 61.31	Φ = 0.35	< 0.001
Psychoanalytical- psychodynamic	13 (13.4)	95 (22.7)			
Humanistic	43 (44.3)	198 (47.4)			
Systemic	9 (9.3)	99 (23.7)			
Behavioral	32 (33.0)	26 (6.2)			
**Gender, n (%)**			χ ² = 1.62	Φ = 0.05	0.445
Women	101 (72.1)	420 (77.2)			
Men	38 (27.1)	120 (22.1)			
Diverse	1 (0.7)	4 (0.7)			
**Age in years, M (SD)**	48.97 (11.23)	52.51 (10.99)	*t*(682) = 3.39	*d* = 0.32	< 0.001
**Professional experience in years** M (SD)	12.90 (9.18)	13.82 (9.78)	*t*(682) = 1.01	*d* = 0.10	0.157

NIG, no interest in VR use group; IG, interest in VR use group based on question “Would you like to use VR for your treatments?”; Percentages are calculated within each column based on the total number of participants in that group.

Differences between women, men and diverse participants were not found. Due to the small number of participants in the diverse group (n=5), a follow-up analysis excluding this group was conducted, which also revealed no significant differences between women and men. Additionally, a t-test indicated that younger professionals were more likely to be interested in VR usage, as evidenced by a significant age difference between IG and NIG participants. Years of professional experience in their respective fields were not different between IG and NIG (see **Table 2**).

### Barriers for non-users of VR in treatment selected in the multiple-choice question

The selected reasons for not adopting VR in treatments are shown in **Table 3**, separately for IG and NIG. In the IG, the most chosen reasons were related to barriers and other causes, whereas the NIG most frequently chose lack of relevance, disinterest, or no perceived advantage as reasons for not using VR.

**Table 3:** Reasons not to adopt VR in treatments: Participant responses by closed answer multiple-choice options.

Reasons	n (%)
IG (n = 140) - Why have you not been able to use VR so far?	
I have reservations about VR so far.	10 (7.1)
I am not sure for which patients VR is beneficial.	18 (12.9)
I’m not sure about the evidence for VR treatments.	21 (15.0)
There are barriers that make it impossible for me to use it.	53 (37.9)
Other	73 (52.1)
NIG (n = 544) - Why is VR not used in your treatments?	
No relevance or not applicable for my patients.	255 (46.9)
Not interested.	256 (47.1)
I don’t see any advantages for me or my patients.	277 (50.9)
I have reservations about VR.	57 (10.5)
There are barriers that make it impossible for me to use it.	61 (11.2)
Other	68 (12.5)

IG, Interested Group; NIG, Not Interested Group. For further detail for the option other or specifications for barriers see qualitative analysis of these responses.

### Barriers to VR adoption: themes from content analysis

**Figure 1:**
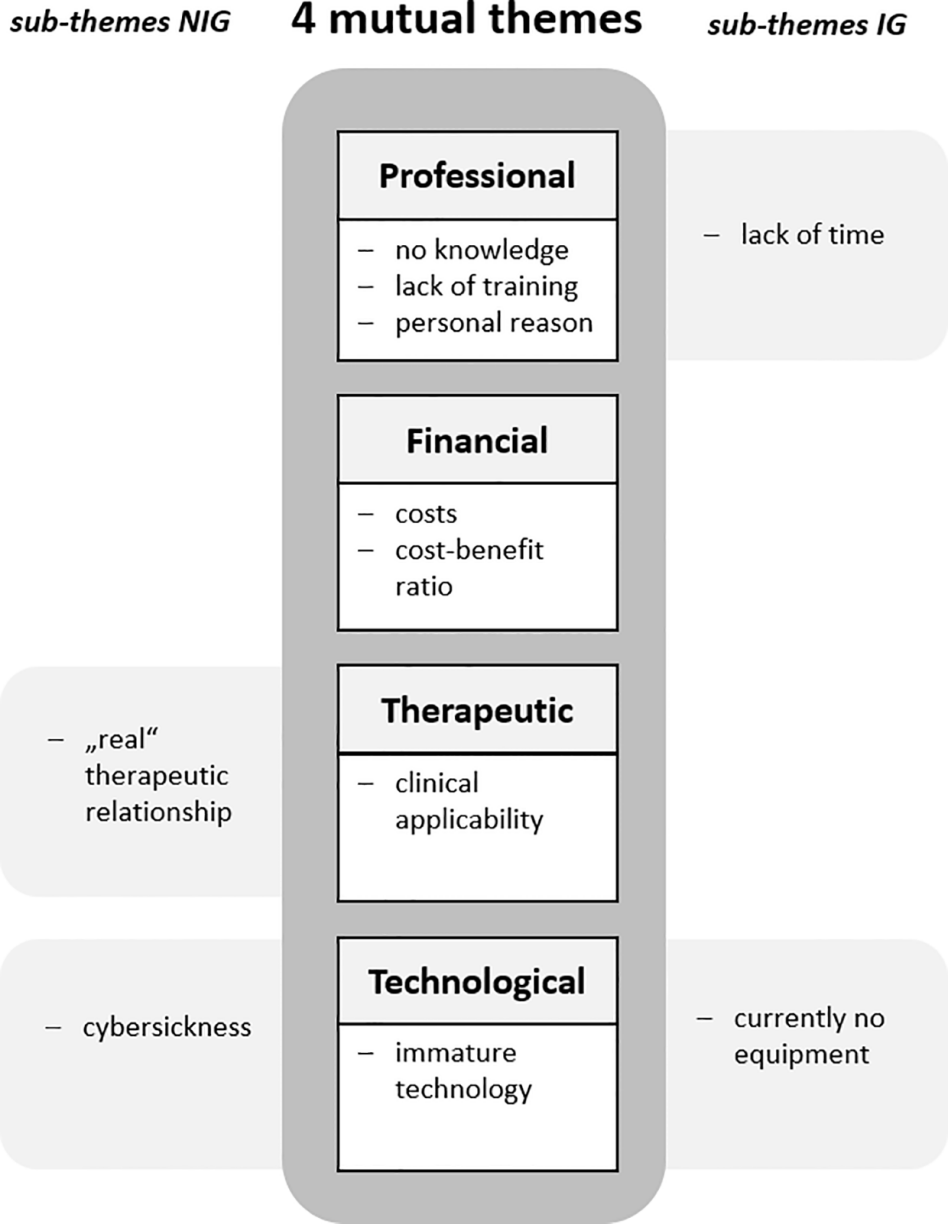
Barriers to VR adoption identified by thematic analysis in Austrian clinical psychologists and psychotherapists. NIG, no interest in VR use group; IG, interest in VR use group based on question “Would you like to use VR for your treatments?”; Presentation of the main topics in descending order by number of responses.

Through inductive content analysis, we identified four broad themes shared by both groups: those not interested in using VR (NIG) and those expressing interest in VR use (IG). Themes are ranked based on the number of responses. They include sub-themes, which are common to both NIG and IG, and some that are specific to either group, see **Figure 1**. The four overarching themes are: (1) *Professional barriers* (with 4 sub-themes), (2) *Financial barriers* (2 sub-themes), (3) *Therapeutic barriers* (2 sub-themes), and (4) *Technological barriers* (3 sub-themes).

#### Professional factors

**Figure 2:**
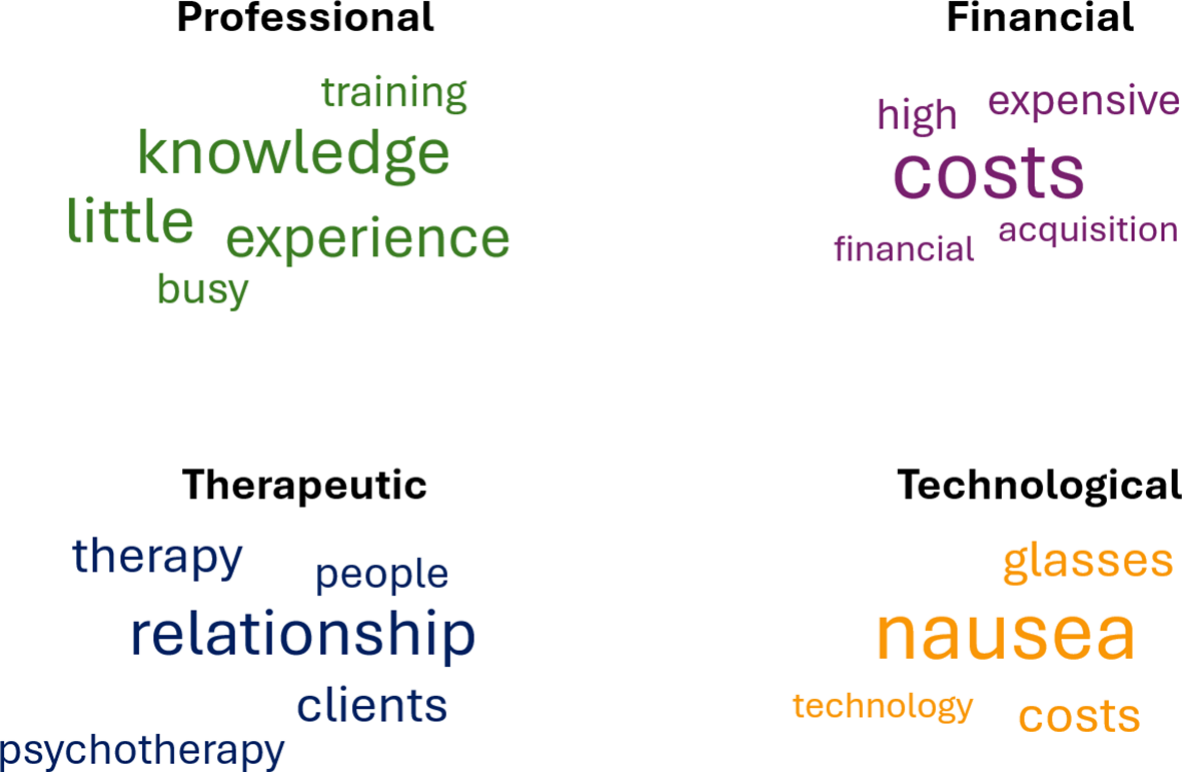
Word cloud representing the four themes identified from the open-ended responses. *Note.* This word cloud was generated using RStudio based on open-ended responses from clinical psychologists and psychotherapists in the online survey. A maximum of five words per theme, each mentioned at least seven times, were selected. The font size reflects the frequency of each word’s occurrence.

The primary and largest theme, based on participants’ responses, centers on professional factors (n=119 responses) related to the therapists themselves, such as their knowledge, training, and attitudes towards VR. Four sub-themes were identified, with the following three shared by both groups (NIG and IG): (1) lack of or limited knowledge (n=59), (2) lack of training (n=33), and (3) personal reasons (n=18). A fourth sub-theme, (4) lack of time (n=9), was specific only to those interested in using VR (IG). For a visual representation of the most frequently cited words per theme see the word-cloud (**Figure 2**).

##### Lack of or limited knowledge

In both the IG and NIG groups, participants reported little to no knowledge of VR technology, citing no prior contact with it and, therefore, being uninformed about relevant scientific evidence. Some participants were entirely unfamiliar with the technology, unaware of its existence or even its use in psychotherapy.

*“I don’t even know what VR means.”* (NIG, m, 43, psychotherapist, humanistic).

*“I’m not familiar with it – neither with the technology itself, nor its practical application.”* (IG, m, 37, clinical psychologist).

Additionally, participants mentioned a lack of or little direct experience with VR, stating that they had not tried it out before and lacked self-experience.

“No knowledge and no self-experience in a therapeutic setting.” (NIG, w, 57, psychotherapist, psychodynamic).

“No experience and no specialist information on this so far.” (IG, w, 34, psychotherapist, psychodynamic).

Participants from both groups also noted that they currently had other priorities and had not yet considered exploring VR for psychotherapy. However, their frequent use of “yet” in their responses suggested they might be open to it in the future.

“I haven’t looked into it yet, so I can’t form an opinion.” (NIG, m, 73, psychotherapist, psychodynamic).

“I haven’t yet thought about offering VR in my treatments. But I am curious and see it as an extension of creative media, especially for working with children and adolescents.” (IG, w, 42, psychotherapist, humanistic).

##### Lack of training

A common theme in both groups was the perceived lack of VR-specific training and further education. At the same time, participants expressed interest in attending relevant courses to expand their knowledge on therapeutic VR.

“No training on this, and too little knowledge of what I can do with it and how.” (NIG, w, 52, psychotherapist, systemic).

“I would like to attend a self-awareness seminar before using the technology. The theoretical seminars I’ve attended so far are not well-founded enough for me.” (IG, m, 52, psychotherapist, humanistic).

##### Personal reasons

Participants from both groups revealed personal reasons that hindered the use of VR in therapy. These included vision impairments, age-related concerns (i.e., feeling too old or approaching retirement), and a perceived lack of technological skills. Additionally, participants in the NIG group specifically cited a lack of motivation for dealing with the subject, feeling that engaging with VR was too cumbersome.

“I’d have to look into it first, but it’s too much of a hassle for me.” (NIG, w, 53, psychotherapist, psychodynamic).

“I myself cannot use VR yet due to my severe visual impairment: I don’t want to use it with my patients without having tried it myself.” (NIG, m, 52, psychotherapist, humanistic).

“I’m just about to retire - and I’m sticking to my way of working.” (IG, m, 61, clinical psychologist).

“I am not very technically savvy.” (IG, w, 52, clinical psychologist, psychotherapist, systemic).

##### Lack of time

A lack of time was exclusively cited by the interest group (IG) as a reason for not yet having adopted VR in therapy.

“Too much else to do so far. Similar to biofeedback, I also think it’s great, but I haven’t had time to learn/acquire it yet.” (IG, m, 33, psychotherapist, behavioral).

“Haven’t had time to look into it in detail yet. In itself, its use in the field of anxiety and obsessive-compulsive disorders would make perfect sense.” (IG, m, 59, psychotherapist, humanistic).

#### Financial factors

The second largest theme, based on participant responses, centered around financial issues (n=85 responses), with both the NIG and IG groups highlighting two sub-themes: (1) costs and (n=72) (2) cost-benefit ratio (n=13).

##### Costs

Participants (both NIG and IG) identified high acquisition costs as well as high running costs as significant barriers to VR adoption. Common themes included the perception that VR is too expensive and that their own financial resources would not permit such an investment. Additionally, a lack of suitable facilities (i.e., rooms) and insufficient funding from external sources were also mentioned as obstacles.

“Financial resources - does not pay off for me in practice alone.” (NIG, w, 29, clinical psychologist).

“No funds in the social sector.” (IG, w, 51, clinical psychologist).

##### Cost-benefit ratio

A number of participants from both groups voiced concerns about the unfavorable cost-benefit ratio of using expensive VR technology in practice. On the one hand, they cited high costs, and on the other hand, they reported an anticipation of low patient demand, an expectation that VR would only be used infrequently, a perception that the technology was too immature, and the belief that anxiety disorders may be more effectively treated with conventional methods, such as *in vivo* exposure.

“In my opinion, the technology is not yet mature enough for use in practice (graphics, price-performance ratio, software, hardware). I see the advantage in exposure work much more in the real setting, without the added value of VR.” (NIG, w, 35, psychotherapist, behavioral).

“High acquisition costs, the patient group for which it makes sense is too small to justify a purchase for me.” (IG, w, 39, clinical psychologist, psychotherapist, behavioral).

#### Therapeutic factors

The third recurring theme centered on therapeutic factors including therapy specific issues (n=69 responses) such as therapeutic approach, patient groups, and professional self-image. A sub-theme shared by both groups (IG and NIG) was (1) clinical applicability (n=46), while a sub-theme including (2) concerns about the “real” therapeutic relationship (n=23) was identified exclusively within the no interest group (NIG).

##### Clinical applicability

Participants from both groups indicated that a potential barrier to implementing VR in psychotherapy was the perceived lack of suitable indication. This perception arose from working with patient populations participants considered unsuitable for VR (e.g., intensive care patients) or from the fact that they were employed in fields (e.g., online counseling) where VR usage is deemed impractical. Additionally, some participants expressed that VR conflicts with their specific therapeutic approaches (e.g., depth psychology or person-centered therapy). Many noted that traditional methods (e.g., hypnosis) are sufficient, and they fear that VR could be a barrier for patients, potentially leading to patient refusal.

“There are therapeutic techniques (hypnotherapy) that can be used. There is no need for VR technology.” (NIG, w, 40, clinical psychologist).

“My psychotherapy method is depth psychological. Virtual glasses would compete with the idea of promoting introspection and finding things there.” (NIG, w, 64, psychotherapist, psychodynamic).

“Still too little relevance for me - so far I have been able to (very) satisfactorily process all aspects of human behavior on a conventional level.” (IG, m, 72, psychotherapist, behavioral).

“Only makes sense if you work with it regularly (more for institutions).” (IG, m, 79, psychotherapist, behavioral).

##### “Real” therapeutic relationship

This sub-theme, which pertains exclusively to the no interest group (NIG), reveals participants’ significant – often emotionally charged – concerns about potential negative effects of VR on the therapist-patient relationship, which was considered as crucial for therapeutic success. Participants expressed that VR – by undermining adequate eye contact and impeding direct person-to-person interaction – could obstruct a “real” and “truthful” encounter between the therapist and the patient.

“Physical eye contact can never be replaced! (NIG, m, 70, psychotherapist, psychodynamic).

“Psychotherapy has the task of enabling people to overcome their real challenges and not to create additional “fantasy worlds”. An essential effective factor in psychotherapy is a healthy therapeutic relationship.” (NIG, m, 65, psychotherapist, systemic).

#### Technological factors

The final theme focused on factors pertaining to the technology itself (n=47 responses), such as its usability and possible adverse effects. This theme included one common sub-theme shared by both NIG and IG (1) immature technology (n=24), and two group-specific sub-themes: (2) cybersickness (NIG, n=10), and (3) lack of equipment (IG, n=13).

##### Immature technology

Participants from both groups (NIG and IG) expressed dissatisfaction with the current state of technology, describing it as immature, uncomfortable, and prone to producing artefacts. They also highlighted the lack of (external) technical support as a concern for using it for psychotherapy.

“No technical assistance.” (IG, w, 54, psychotherapist, humanistic).

“Technology is not yet good enough. Scenarios are not realistic enough and there is too little variance in the scenarios.” (NIG, w, 34, clinical psychologist).

##### Cybersickness

Only the no interest group (NIG) raised concerns about cybersickness as a potential side effect of VR. Some participants reported experiencing vertigo and nausea themselves when using VR, while others expressed worries about their patients potentially facing similar adverse reactions.

“Triggers dizziness, nausea, vomiting and headaches for me.” (NIG, w, 58, psychotherapist, psychodynamic).

“Kinetic nausea in about 50% of users.” (NIG, w, 40, clinical psychologist, psychotherapist, behavioral).

##### Lack of equipment

In contrast to NIG, participants from the interest group (IG) identified the absence of VR equipment as a key barrier to its adoption. They explained that not having access to this technology currently prevents them from incorporating VR into their practice.

“No suitable VR glasses and software currently available.” (NIG, w, 39, clinical psychologist).

“I don’t have VR glasses and am not yet familiar with the technology.” (NIG, w, 42, clinical psychologist).

## Discussion

These professional, financial, therapeutic, and technological barriers highlight the difficulty of transferring evidence-based interventions into clinical practice for Austrian psychotherapists and clinical psychologists. To our knowledge, this is the first study to assess the rate of therapeutic VR adoption among a large, diverse sample of practicing Austrian clinical psychologists and psychotherapists. In addition to identifying group differences between mental health professionals interested and not interested in using therapeutic VR, this comprehensive online survey aimed to explore contextual and individual barriers to VR adoption in psychotherapy. Consistent with previous findings (26, 27, 30, 34), the reported rate of VR adoption for therapy was very low (1%) among participating clinical psychologists and psychotherapists. Similar surveys report that 0% of a sample of Australian mental health care workers have used VR in their practice (30), while 14% of mental health professionals at the 2016 European Association of Behavioral and Cognitive Therapies conference in Sweden reported having done so (26). This contrasts sharply with the growing body of evidence supporting VR’s effectiveness in treating various mental disorders (e.g., 1) and highlights the slow, challenging process of integrating evidence-based methods into clinical practice (Grol and Grimshaw, 1998; 33). Similarly, only 20.5% of participants expressed interest in using VR for therapeutic purposes in the future (interest group, IG), while 79.5% indicated no intention to adopt it (no interest group, NIG), reflecting low intrinsic motivation and even resistance to using VR in therapy.

### Group differences between those interested in using VR for treatment and those who are not interested

Austrian mental health professionals with prior VR experience were more interested in applying it clinically than those who had never tried VR themselves. This aligns with previous findings from international behavioral therapists on VR exposure therapy, where prior VR experience predicted future likelihood of its use (26). It is also consistent with studies on other technology-supported treatment options, such as teletherapy in Austria, where experience with the technology led to greater acceptance of its use (43). To benefit from this effect, VR could be offered in education and training to give therapists and clinical psychologists the opportunity to familiarize themselves with it.

Furthermore, our results indicate a greater interest in VR usage among psychotherapists and clinical psychologists who are employed or both self-employed and employed, compared to those solely in self-employment. This pattern may reflect the advantages associated with employment, such as easier access to financial resources and technical support, which helps reduce the individual burden on mental health practitioners and fosters a greater openness to integrating VR in clinical practice as prior research suggests that financial and technical barriers are major obstacles in adopting VR in clinical settings (26, 31, 34, 44). This trend aligns with our finding that professionals with training in clinical psychology or both clinical psychology and psychotherapy showed more interest in VR for clinical use than psychotherapists alone, as clinical psychologists typically work in clinics and institutions in Austria, whereas psychotherapists are self-employed in private practice more frequently. This context may contribute to greater openness among clinical psychologists toward adopting new technology in their clinical work. To encourage VR adoption, both institutional and individual practitioners could benefit from reduced costs and improved usability of VR technology. While clinical psychologists in Austria show more interest to increasingly embrace technology, the interest in adopting VR technology appears more nuanced among psychotherapists, varying significantly by therapeutic orientation.

Among psychotherapists in our sample, those trained in behavioral therapy demonstrated a stronger interest in VR adoption, whereas psychoanalytic-psychodynamic and systemic psychotherapists showed less interest in VR usage. Similar tendencies were found for the therapeutic orientation in research on other technology like web-based therapy in psychotherapy with negative attitudes in psychodynamic therapists (45) and positive ratings and associations in behavioral therapists (26, 46). This may not only reflect differing attitudes toward VR, but also VR’s strong applicability in exposure therapy – a core behavioral technique – where good effects of VR were reported (8, 13, 47). By contrast, VR may be less compatible with approaches focused on interpersonal dynamics and self-exploration, which are central to psychodynamic and systemic therapies, as some participants noted in their comments (see qualitative analysis). The development of additional VR applications, such as relationship therapy, mindfulness, safe place, relaxation exercises, and play therapy with role-playing elements – as requested by mental health therapists in the U.S. (44) – could support a more widespread use across diverse therapeutic approaches in the future.

A small but significant age effect was found, with younger therapists showing more interest in VR. This might be due to feeling less capable or confident in this area as it was found in a study on child and adolescent mental health services in the UK, where older clinicians reported feeling less skilled and competent (48). Finally, while we observed differences in VR interest based on experience with VR, employment type, clinical training, therapeutic orientation, and age, we found no gender-based differences or differences of therapeutic/clinical experience. While some studies indicate that men may be more inclined to use digital medical applications, as seen in a Dutch study of elderly individuals (49), and other research suggests that women possess more knowledge about technology for caregiving (50), our findings did not reveal any significant differences between genders in terms of interest in VR usage among mental health professionals. This is in line with previous research suggesting similar technology adoption rates between genders in clinicians (51) and no association of gender with perceived benefits or costs of VR use in a study on practicing psychotherapists in Canada (52). Thus, despite varying gender-related findings in specific contexts, our study aligns with the broader trend of equal interest in technology adoption across genders in clinical settings.

### Barriers to implementation

Results of the multiple choice questions concerning reasons why VR is not used in treatments suggest that the IG tends to be more concerned with practical issues related to VR implementation as they chose “There are barriers that make it impossible for me to use it.” and “Other” most often, while the NIG views VR as having no advantages, not beneficial or irrelevant to their treatments.

A more in depth thematic analysis of open responses, identified four main barriers to VR implementation shared by both groups (IG and NIG): *professional barriers* (sub-themes: lack of knowledge, training and time, as well as personal reasons), *financial barriers* (costs, cost-benefit ratio), *therapeutic barriers* (clinical applicability, concerns about the “real” therapeutic relationship), and *technological barriers* (immature technology, cybersickness, lack of equipment). To interpret these themes and recommend suitable interventions, we will use the *Theoretical Domains Framework* (TDF; 53), which offers a structured approach for assessing implementation challenges, in combination with the *Behavior Change Wheel* (BCW; 54). The BCW identifies three essential preconditions for behavior change (*capability, opportunity, motivation – behavior change*, *COM-B*): (1) *capability*, the individual’s ability to engage in the intended behavior; (2) *opportunity*, external factors that facilitate or hinder behavior change; and (3) *motivation*, encompassing processes such as habits, emotions, and decision-making relevant for behavior change. Resulting interventions and policies are based on this system and specifically address deficits in the above-mentioned preconditions.

#### Capability

Both the interest group (IG) and no interest group (NIG) reported significant gaps in knowledge about VR in therapy, including a lack of understanding of existing evidence, with some participants even being completely unfamiliar with the term “VR” (26, 30, 34). This is somewhat surprising given the increased exposure of the public to VR technology since the release of the Oculus Rift in 2016. In addition, both groups also reported lacking the necessary skills, experience, and training to implement VR (see *professional* theme), all of which are an integral part of behavior change according to TDF and BCW. This limited knowledge of VR’s applicability has important implications. Participants (see *therapeutic* theme) expressed uncertainty about its appropriateness, associating VR primarily with exposure therapy and anxiety disorders, while other applications seemed to have less intuitive validity (34). Many felt VR was unsuitable for their therapeutic approach, their patient population, or their field of work. This misconception of what therapeutic VR may be used for (e.g., 1), likely also influenced perceptions of the cost-benefit ratio (*financial* theme); without a clear understanding of VR’s potential, its usefulness for participants’ practice was undervalued. Addressing these issues based on BWC requires introducing comprehensive education programs as early as in university curricula to enhance familiarity with current evidence and best practices. Reinforcing clinical guidelines and offering specialized training to build practical skills would further support the adoption of VR in therapy.

#### Opportunity

In line with previous research (31, 34), both groups (IG and NIG) frequently cited high acquisition and maintenance costs (*financial* theme) as a barrier to using VR. Additionally, participants criticized the lack of reimbursement for VR-based therapies and inadequate workplace support for implementing the technology. While most responses did not specify whether the issue was hardware or software costs, some pointed to the contrast between affordable, accessible hardware (HMDs) and the lack of, or too expensive software. Low-quality software, limited commercial scenarios, and a lack of reliable providers further seems to discourage VR adoption. Even those who have already adopted VR (1% of VR users in this study) expressed a need for better quality, more user-friendly software and increased technical support. Both NIG and IG perceived the technology as immature – bulky, low-quality, and cumbersome – indicating that technological advancements have not fully addressed those barriers which were voiced in the early 2000s (26). Therefore, companies developing hardware and software for these purposes could continue to improve the quality of their offerings like personalization, complexity and interactivity through higher computational power and more precise physical movement- and eye-tracking (55). To avoid or reduce VR-induced symptoms and effects like cybersickness, developers but also clinicians may consult the guidelines suggested by Souchet et al. (56) for future developments and decisions. Providers could also consider offering therapists and clinical psychologists the opportunity to test their products to the latest standards, since hands-on experiences with the latest products could help convince potential users who may be skeptical about recent advancements. Collaboration with professionals could also ensure that the technology aligns with real-world clinical needs, enhances user experience, and meets the standards in therapeutic settings.

Another common constraint, especially among the interest group, was time, with many participants citing other professional obligations as limiting their ability to explore VR. To encourage sustainable adoption of therapeutic VR, future efforts should focus on incentivizing its use in scenarios where traditional *in-vivo* exposure therapy is challenging or costly, for example with anxiety related to thunderstorms or flights. In these cases, VR can be an accessible and effective alternative, providing a safe, controlled environment, adding an expansion of therapeutic intervention options. Incentives could include providing reimbursements for VR technology in the public health sector, funding software development, offering effective technical support for VR users, and encouraging organizations to invest in the necessary infrastructure and personnel for administering VR treatments. Furthermore, clinical decision-making frameworks should be developed to guide the selection of appropriate VR systems based on therapeutically relevant characteristics (e.g., 57).

#### Motivation

The largest category related to participants’ intention to use VR, encompassed their professional identity, beliefs about their own capabilities, and emotional reactions. Most participants (79.5% NIG) were not interested in VR, and only 20.5% expressed an intention to use it. This reluctance was often driven by misconceptions about VR’s role in the therapeutic process (*therapeutic* theme), such as the belief that it obstructs face-to-face interaction and hinders the patient-therapist relationship. Given psychotherapy’s traditional focus on human-to-human interaction (27), this concern is common (31), despite evidence disproving it (58). Some participants may have perceived VR as a threat to conventional approaches (59), which was reflected in their highly emotional responses, such as referring to VR as “absurd” or “showmanship”. VR was also seen as conflicting with professional identity and personal abilities. Some participants felt too old or not tech-savvy enough to adopt VR (*professional* theme), while others expressed concerns about their own tolerability of the technology, including vision issues or susceptibility to cybersickness (*technological* theme). In some cases, participants overestimated the adverse effects of VR on patients. Addressing these motivational barriers, particularly common misconceptions about VR’s role in the therapeutic process, may require specialized, multi-stage training (60, 61), beginning with general knowledge and progressing to clinical skills. This could help clarify VR’s benefits and limitations for therapy (34), reducing fears that it threatens traditional therapeutic approaches. Additionally, fostering social connections with clinicians who successfully use VR, and engaging practitioners in research (35) may also help enhance motivation through positive social influence.

### Strengths and limitations

To our knowledge, this is the first study to assess a large, diverse sample of active clinical psychologists and psychotherapists regarding their current use of therapeutic VR, as well as the contextual and individual barriers to its adoption in Austria. We employed an inductive content analysis approach, allowing the data to speak for itself rather than using a pre-existing theoretical framework. However, by linking our findings to two established behavior change frameworks (the TDF and BCW), we were able to situate our findings within the broader field of implementation research and draw more robust conclusions about necessary interventions and policies.

Despite these strengths, our results must be interpreted in light of limitations. We conducted an online survey, which led to incomplete responses, particularly with some participants providing only brief or keyword-based answers to open-ended questions, making it difficult to fully interpret their intent. Furthermore, for exploratory reasons we did not use standardized questionnaires to assess VR usage, knowledge of VR and attitudes towards VR. Also, we did not reach out to those currently training to become a clinical psychologist or psychotherapist to assess the current state of the art regarding VR in training. Results on current VR usage should also be interpreted with caution, as only 10 participants indicated using VR for therapeutic interventions, limiting the generalizability of these findings. Future research could benefit from drawing smaller but still representative samples from the VR non-user group and a larger sample of the VR users and conducting in-depth interviews with them, to further explore the nuanced interplay of contextual and individual barriers to VR adoption in psychotherapy. Also, future studies should include the perspective of the patients to complement the results found for mental health practitioners. Prior research indicates that patients prefer VR- exposure over *in-vivo* settings (28, 29). Especially fearful patients showed higher preference and acceptance for VR compared to *in-vivo* exposure which might lower refusal rate for this patient group (Scheveneels et al. (62). Future research could furthermore explore the sources clinicians rely on for information about therapeutic VR. While this study did not assess that aspect, understanding whether different sources (e.g., scientific papers, public media, peers) influence clinicians’ attitudes toward VR could help inform the design of targeted training programs.

## Conclusion

In conclusion, our study highlights significant barriers to the adoption of therapeutic VR among Austrian clinical psychologists and psychotherapists, encompassing, on the one hand, individual aspects (see BCW precondition *capability*), contextual factors (*opportunity*), and emotional aspects and habits (*motivation*). Both the interest group (IG) and no interest group (NIG) voiced considerable potential to build knowledge and skills related to effective implementation of therapeutic VR. These limitations in factual knowledge and VR skills, linked with widespread misconceptions regarding the actual role of VR in therapy, led to an underappreciation of its widespread potential benefits. Financial constraints, high costs, lack of reimbursement, and insufficient workplace support further hindered VR adoption, with many participants (both NIG and IG) perceiving the technology as immature and cumbersome. Motivational barriers, such as concerns about VR undermining the therapeutic relationship, resistance to new technologies, and personal limitations (e.g., age, tech-savviness), were also prevalent.

To promote wider use of therapeutic VR, dissemination and implementation efforts should focus on providing comprehensive education as well as specialized training programs including opportunities to familiarize oneself with VR technologies and on developing specified clinical guidelines for therapeutic VR usage. These trainings could include online courses and introductory workshops covering the basics of VR technology, as well as hands-on training with immersive demonstrations and supervised practice sessions with case studies to build confidence in using VR. Also, mentorship programs pairing VR novices with experienced clinicians as well as online forums for sharing experiences may support the successful implementation of VR in clinical practice. Furthermore, facilitating financial support for VR technologies and supporting the development and implementation of specialized VR software beyond exposure therapy, as well as introducing reimbursements in the public health sector for VR treatments, and fostering a positive social influence through peer engagement, could all help to fully exploit the great potential of VR for mental health disorders.

## Data Availability

The raw data supporting the conclusions of this article will be made available by the authors, without undue reservation.
