# Supplementary material for: Barriers to adopting therapeutic virtual reality: the perspective of clinical psychologists and psychotherapists
Source: Front Psychiatry. 2025 Mar 18;16:1549090. doi: 10.3389/fpsyt.2025.1549090 (PMC11958971; doi:10.3389/fpsyt.2025.1549090)
Supplement: Supplementary file 1 [file SupplementaryFile1.docx]

**Supplement A. Online questionnaire and item wording**

**Do you use virtual reality (VR) in your treatments (via VR glasses, e.g. Meta Quest)?**

- Yes/ No

IF YES:

**For which patients do you use VR?** (Selection - several possible)

- for children/adolescents
- for adults
- Anxiety disorders (which ones? - free text)
- depression
- Eating disorders
- PTSD
- ADHD
- ASD
- Schizophrenia
- Somatic diseases (which ones? - free text)
- Other: (free text)

**In your opinion, what are the advantages of using VR?**

- for you? (free text)
- for the patients? (free text)

**What problems does it cause?**

- for you? (free text)
- for the patients? (free text)

**What would you wish for the implementation of VR in treatment?** (free text)

IF NO:

**Have you ever tried VR glasses yourself?**

- Yes/ No

**Would you like to use VR for your treatments?**

- Yes/ No

**If no (no interest group, NIG): What are the reasons for not wanting to use VR?** (Selection - several possible)

- No relevance/not applicable for my patients
- No interest
- Do not see any benefit for me/for my patients
- I have reservations about VR – which ones? (free text)
- There are barriers that make it impossible for me to use it – which ones? (free text)
- Other reasons – which ones? (free text)

**If yes (interest group, IG):** Why haven’t you been able to use VR yet? (Selection - several possible)

- I am not sure for which patients VR is beneficial
- I am not sure about the evidence for VR treatments
- I have reservations about VR – which ones? (free text)
- There are barriers that make it impossible for me to use it – which ones? (free text)
- Other reasons – which ones? (free text)
